# mTOR: Alzheimer's disease prevention for APOE4 carriers

**DOI:** 10.18632/oncotarget.10349

**Published:** 2016-06-30

**Authors:** Ai-Ling Lin, David Allan Butterfield, Arlan Richardson

**Affiliations:** Sanders-Brown Center on Aging, Department of Pharmacology and Nutritional Sciences, University of Kentucky, Lexington, Kentucky, USA; Department of Biomedical Engineering, University of Kentucky, Lexington, Kentucky, USA

**Keywords:** rapamycin, mTOR, Alzheimer's disease, cerebral blood flow, blood brain barrier

Alzheimer's disease (AD) is the most common form of dementia and the 6th leading cause of death in the US. Inheritance of the Apolipoprotein ε4 (APOE4) allele is the strongest genetic risk factor for late-onset AD - APOE4 carriers accumulate beta-amyloid (Aβ) and neurofibrillary tau tangles earlier and with more extensive pathology compared to non-carriers. However, decades before the aggregation of Aβ and tau, cognitively normal APOE4 carriers have developed neurovascular deficits, including reduced cerebral blood flow (CBF) and impaired bloodbrain barrier (BBB) integrity [1]. The above indicates that brain physiology is altered in APOE4 carriers before clinical markers such as Aβ, tau pathology, and memory deficits appear, suggesting that vascular changes predispose APOE4 carriers to developing AD. Therefore, early interventions that can restore neurovascular deficits to normal could be critical in potentially preventing the development of AD-related neuropathology and cognitive impairment. In a recent study, using magnetic resonance imaging (MRI), *Lin et al.* reported that young, asymptomatic APOE4 targeted-replacement mice treated with rapamycin had restored CBF and BBB integrity to the level of wild-type controls [2]. The preserved vasculature was associated with amelioration of incipient learning deficits of the APOE4 transgenic mice. The significance of this discovery is that brain functions are pharmacologically reversible and AD potentially could be prevented in asymptomatic APOE4 carriers.

Rapamycin is a drug approved by US Food and Drug Administration (FDA). It has been widely used in clinical settings and was originally used as an immunosuppressive agent to prevent rejection of organs in transplant patients. By 1991, Heitman and co-workers discovered a kinase, Target of Rapamyin (TOR) [3]. In mammals, the protein is specified as mammalian TOR, or mTOR. mTOR is the serine/threonine kinase that is the regulatory nexus in the response of eukaryote cells to nutrients, growth factors, and cellular energy status. Rapamycin is a mTOR inhibitor. A major breakthrough occurred in 2009 when it was shown that rapamycin, which reduced mTOR signaling, increased the lifespan of mice (see review in [4]). In addition, rapamycin has been shown to reduce a variety of cancers in mice as well as atherosclerosis in mouse models fed high fat diets as well as improve immunity in elderly humans [5]. As regard to the human central nervous system, several studies have shown that inhibition of mTOR by rapamycin treatment improves cognition, slows brain aging, and impedes the progress of neurodegenerative disorders through pathways associated with autophagy, glucose metabolism and mitochondrial functions [6].

*Lin et al.* demonstrated that mTOR inhibition reduced proinflammatory pathways in brain vasculature that otherwise impair BBB integrity in the APOE4 transgenic mice [2]. Loss of BBB integrity is considered one of the initiating events that lead to AD-like pathological cascade in the APOE4 carriers [1]. Restoring BBB integrity is highly associated with improved CBF and preserved learning ability of the APOE4 transgenic mice [2]. In addition, *Lin et al.* previously reported that inhibition of mTOR activates endothelial nitric oxide synthase and causes the release of nitric oxide, a vasodilator, which in turn increases CBF [7]. In mice modeling human AD (hAPP (J20)), *Lin et al.* found that rapamycin restored their CBF and vascular density, which were associated with reduced accumulation of Aβ and cerebral amyloid angiopathy, and improved memory [7]. Collectively, these findings indicate that rapamycin alters neurovascular functions through multiple potential pathways. Because neurovascular defects are one of the earliest events that lead to AD-like pathology, mTOR inhibition might be an effective intervention to preserve brain vascular functions and consequently slow or prevent the progress of AD development.

In conclusion, mTOR inhibition has been shown to increase lifespan and healthspan in various species. Rapamycin (or its derivatives, rapalogues) has been approved by the FDA since 1999 for various uses in humans, and these compounds have been given to cancer patients for relatively long periods of time with little change in the quality of life. *Lin et al.* further demonstrated that rapamycin restores and preserves neurovascular functions in the APOE4 transgenic mice using MRI. Their results may provide the basis for future AD prevention trials in human APOE4 carriers.
